# Chitosan-Coated Fe_3_O_4_ Nanoparticles for Magnetic Hyperthermia

**DOI:** 10.3390/ma18245629

**Published:** 2025-12-15

**Authors:** Aleksandra Wilczyńska, Leszek Ruchomski, Mateusz Łakomski, Małgorzata Góral-Kowalczyk, Zbigniew Surowiec, Arkadiusz Miaskowski

**Affiliations:** 1 Department of Electronics and Information Technology, Lublin University of Technology, 38A Nadbystrzycka Street, 20-618 Lublin, Poland; a.miaskowski@pollub.pl; 2 Department of Electrical Engineering and Intelligent Technologies, Lublin University of Technology, 38A Nadbystrzycka Street, 20-618 Lublin, Poland; l.ruchomski@pollub.pl; 3 Department of Semiconductor and Optoelectronic Devices, Lodz University of Technology, 8 Politechniki Ave, 93-590 Łódź, Poland; mateusz.lakomski@p.lodz.pl; 4 Department of Agricultural, Forestry and Transport Machines, University of Life Sciences in Lublin, 13 Akademicka Street, 20-950 Lublin, Poland; malgorzata.goral-kowalczyk@up.lublin.pl; 5 Institute of Physics, Maria Curie-Sklodowska University, 1 Maria Curie-Skłodowska Sq., 20-031 Lublin, Poland; zbigniew.surowiec@mail.umcs.pl

**Keywords:** magnetite nanoparticles (Fe_3_O_4_), ferrofluids, electromagnetic properties, electrokinetic properties, impedance spectroscopy, calorimetry, x-ray diffraction, Mössbauer spectra, SEM-EDS analysis

## Abstract

This work investigated the electrical, dielectric, and magnetic properties of ferrofluids containing Fe_3_O_4_ nanoparticles and their composites with chitosan (30–100 cP and 100–300 cP), relevant to magnetic hyperthermia. The nanoparticles were synthesized by coprecipitation and characterized using impedance spectroscopy, X-ray diffraction, scanning microscopy with X-ray microanalysis, Mössbauer spectroscopy, and calorimetry. The study showed that the chitosan coating altered the textural properties of Fe_3_O_4_, reducing the specific surface area from 76.3 m^2^/g to 68.9–72.5 m^2^/g. The zeta potential and particle size showed strong pH dependence. Impedance measurements showed that the conductivity of ferrofluids was frequency- and temperature-dependent, with both metallic and dielectric conductivity observed. The complex dielectric permittivity exhibited Maxwell–Wagner–Sillars interface polarization. Calorimetry revealed that specific absorption rate (SAR) ranged from 11.4 to 23.4 W/g, depending on the chitosan concentration and type, while the chitosan coating reduced SAR by 12–40%. These results confirm that the electrical and dielectric parameters of ferrofluids significantly influence their thermal capabilities, which is important for optimizing magnetic hyperthermia therapy when energy dissipation is considered in bio-heat models.

## 1. Introduction

Medical nanotechnology is currently experiencing rapid development in the field of cancer therapy, particularly in the context of magnetic hyperthermia as an innovative treatment method. Ferrofluids containing Fe_3_O_4_ nanoparticles represent a promising class of biomedical materials that combine unique magnetic properties with the ability to precisely control the temperature in tumor tissues when exposed to an alternating magnetic field [1,2].

Fe_3_O_4_ (magnetite, ferrite) is a material exhibiting superparamagnetic properties, which make it suitable for numerous applications. Interest in Fe_3_O_4_ nanoparticles has increased significantly in recent years due to their properties. In biomedical applications, Fe_3_O_4_ has shown promising results in magnetic hyperthermia (MHT) for cancer treatment, as a contrast agent in Magnetic Resonance Imaging (MRI), and in targeted drug delivery. In catalysis, Fe_3_O_4_ and its nanocomposites are used in organic transformations and photocatalysis. Adding elements allows for further optimization of structural and magnetic properties [3,4,5].

The effectiveness of this therapy is determined by many factors like position and shape of the tumor, the strength of the magnetic field and its amplitude and concentration, and the type of magnetic nanoparticles used during therapy. All these factors can be controlled during treatment planning, but in order to do so, all the aforementioned factors should be precisely recognized in vitro or in silico. When it comes to magnetic nanoparticles (MNPs) as such, their heating ability is quantified by the specific absorption rate (SAR), which measures the amount of energy absorbed per unit mass of magnetic material per unit time. SAR also depends on many factors, including the magnetic field amplitude and its frequency, the magnetic properties of the nanoparticles, and the electrical and dielectric properties of the medium in which the nanoparticles are located [6,7,8,9].

Despite the rapid development of research on magnetic nanoparticles and their application in hyperthermia, the electrical and dielectric characterization of ferrofluids remains a relatively underexplored area. Most studies focus on the magnetic and thermal properties of nanoparticles, while systematic studies of electrical parameters, such as conductivity and dielectric permittivity, of ferrofluids intended for biomedical applications are insufficient. This knowledge gap is particularly significant because electrical and dielectric properties are crucial for the precise modeling and treatment planning of magnetic hyperthermia therapy. Knowledge of the electrical and dielectric parameters of ferrofluids is essential for solving, for example, the Pennes bioheat equation in silico, which is a fundamental mathematical model describing temperature distribution in biological tissue during thermal therapy. In this case, the electrical conductivity of ferrofluids and tumor tissues is a key parameter when analyzing electromagnetic field distribution, as the distribution of the magnetic field together with frequency determines the SAR value [10]. On the other hand, considering calorimetric experiments, it is assumed that the power is deposited by MNPs, but in practice, the recorded temperature rise is not only due to magnetic losses but also due to conductive and dielectric losses.

In this context, the analysis of changes in dielectric properties allows for a deeper understanding of the physical processes in ferrofluids. The interaction of the electric field and magnetic flux density affects the dielectric parameters and the arrangement of the magnetic nanoparticles in the ferrofluid. This phenomenon can be studied using dielectric spectroscopy [11].

Impedance spectroscopy is an advanced electrical characterization technique that is gaining increasing importance in the study of ferrofluids based on Fe_3_O_4_ nanoparticles dispersed in water. This method enables comprehensive analysis of the charge transport mechanisms, dielectric polarization, and relaxation processes occurring in these complex colloidal systems [12]. This spectroscopy involves the application of a low-amplitude sinusoidal signal to the ferrofluid under investigation over a wide frequency range (from Hz to MHz) and measuring the system response. In aqueous ferrofluids containing Fe_3_O_4_ nanoparticles, impedance spectroscopy allows for the identification of several characteristic charge transport mechanisms, i.e., electrical conduction in MNP nanoparticles occurs via electron hopping between Fe ions, which is particularly evident at low frequencies. This hopping mechanism is thermally activated and exhibits a characteristic temperature dependence consistent with the Arrhenius law. Furthermore, Maxwell-Wagner-Sillars interfacial polarization occurs at the interface between the conducting nanoparticles and the insulating aqueous medium, leading to charge accumulation at the interfaces. Ionic conduction in the aqueous phase of the ferrofluid, associated with the presence of ions from the synthesis processes and surfactants, particularly influences the low-frequency characteristics [13,14,15].

The significant conductivity variation with frequency is due to significant changes in the particle diameter. Calculations have shown that the electrical parameters of iron(III) oxide (Fe_2_O_3_) nanopowder exhibit a strong frequency dependence [16]. Similarly, the conductivity of ferrofluids, such as hematite (α-Fe_2_O_3_), is consistent with the mechanism of electron hopping between nanograins [17,18].

The dielectric behavior of colloidal systems consisting of nanoparticles suspended in a carrier liquid like water can be theoretically described by the Schwarz model, where an alternating electric field causes deformation of the ionic atmosphere surrounding the particles, which leads to their polarization [19].

Studies on the electrical properties of ferrofluids, such as their electrical conductivity, are still limited. In [13], the authors determined the dependence of electrical conductivity σ(*f*, *T*) on frequency and temperature. In [20], the impedance behavior of two ferrofluids with a similar magnetic composition was analyzed—one with spherical nanoparticles and the other composed of cubic nanoparticles suspended in kerosene. 

## 2. Materials and Methods

### 2.1. Synthesis and Characterization of Fe_3_O_4_ and Fe_3_O_4_/Chitosan Composites

All the chemical reagents were purchased from commercial suppliers and used for synthesizing composites without any purification. For the synthesis of composites, the materials used were iron(III) chloride hexahydrate (FeCl_3_∙6H_2_O, Pol-Aura, Morąg, Poland, iron(II) chloride tetrahydrate (FeCl_2_∙4H_2_O, J.T.Baker, NJ, USA, ammonium hydroxide solution (NH_3_∙H_2_O, 25%, POCH, Gliwice, Poland, acetic acid (CH_3_COOH, 99.5%, Warchem, Zakręt, Poland, chitosan 30–100 cP (Pol-Aura, as provided by the manufacturer, labeled as “CPS”), chitosan 100–300 cP (Pol-Aura, as provided by the manufacturer, labeled as “CPS”), dichloromethane (CH_2_Cl_2_, Chempur, Brzeg Dolny, Poland, deionized water (Hydrolab, Straszyn, Poland), and nitrogen gas.

The synthesis of Fe_3_O_4_ was performed following published procedures, which were modified and tailored to the requirements of our study [21,22].

Into the reactor, 4.30 g of FeCl_2_·4H_2_O, 11.80 g of FeCl_3_·6H_2_O (with a molar ratio of FeCl_2_·4H_2_O to FeCl_3_·6H_2_O of 1:2), and 400 mL of deionized water were introduced. The reaction was conducted under magnetic stirring at 80 °C, while the mixture was purged with nitrogen. Subsequently, 45 mL of ammonium hydroxide solution (25% NH_3_·H_2_O) was slowly added dropwise, adjusting the pH of the mixture to approximately 10. The precipitated Fe_3_O_4_ was washed with chilled deionized water and separated using a neodymium magnet. The obtained product was dried at 60 °C.

A total of 1.5 g of Fe_3_O_4_ was dispersed in 50 g of distilled water using ultrasonic treatment, and 0.75 g of chitosan was dispersed separately under the same conditions. The chitosan dispersion was then added dropwise to the Fe_3_O_4_ suspension, and the reaction was carried out at 60 °C under magnetic stirring. The resulting composite was separated using a neodymium magnet, washed with CH_2_Cl_2_, and dried at 60 °C. Following this procedure, two materials were prepared using chitosans of different viscosities. Specifically, chitosan 30–100 cP (average molecular weight 250,000) was used to obtain Fe_3_O_4_/chitosan (30–100 cP), and chitosan 100–300 cP (average molecular weight 890,000) was employed to prepare Fe_3_O_4_/chitosan (100–300 cP). Very viscous chitosan solutions limit diffusion and can hinder uniform coating of Fe_3_O_4_ surfaces, leading to heterogeneous shell formation and compromised steric stabilization. Therefore, while moderate increases in molecular weight improve nanoparticle stabilization, there exists a practical upper limit—typically in the range of a few hundred cP—beyond which further increases in viscosity may destabilize the dispersion rather than improve it.

All materials were characterized by specific surface area (SSA, BET), volume of pores, micropore volume, and average pore width. The specimens (35 mg) were dried for 2 h at 70 °C prior to the measurements (Gemini V, Micromeritics, Norcross, GA, USA).

The zeta potential (calculated using the Smoluchowski equation) and particle size (conducted using Dynamic Light Scattering-DLS) as a function of pH were measured in 1:10,000 (*w*/*w*) dispersions prepared in 1 mM NaCl solution at 25 °C and polydispersity index (PDI) using a Malvern Zetasizer (Malvern, Worcestershire, UK). Zeta potentials were measured for aqueous dispersions across the pH range of 2–11. pH adjustments were performed by adding small volumes of diluted HCl or NaOH solutions directly to the dispersions before measurement.

### 2.2. X-Ray Diffraction Measurements

The powder XRD diffraction patterns were recorded at room temperature using a PANalytical X’Pert Pro (PANalytical B.V., Almelo, The Netherlands) diffractometer equipped with a Cu Kα1 radiation source (λ = 1.54059 Å) operating in a standard θ–2θ geometry. The phase and structure analyses were performed using the PANalytical X’Pert High ScorePlus version 2.2.1 software with the ICDD PDF database. The Williamson–Holl method [23] was used to determine the size of crystallites and lattice strain. An NIST CeO_2_ standard was used to correct for instrumental broadening and verify the results.

### 2.3. Measurements of the AC Properties of Ferrofluids

**Figure 1:**
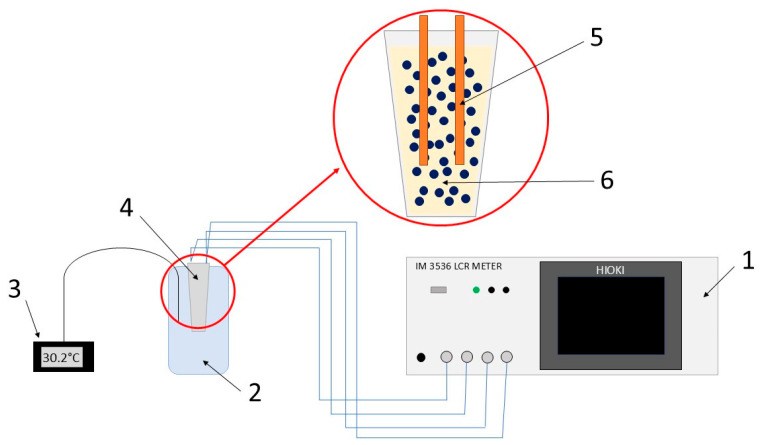
Schematic diagram of a measuring station for the electrical properties of ferrofluids. It consists of the following components: 1—Hioki 3536 impedance meter; 2—vessel with water; 3—temperature meter; 4—Eppendorf; 5—electrodes; 6—ferrofluid.

Fe_3_O_4_ nanoparticles were dispersed in distilled water. Two samples were prepared for each of the three types of nanoparticles (without chitosan, with chitosan at a viscosity of 30–100 cP, and with chitosan at a viscosity of 100–300 cP): the first at a concentration of 100 mg/1 mL of water, the second at a concentration of 50 mg/1 mL of water, and the third at a concentration of 25 mg/1 mL of water. The same suspensions were subjected to electrical and dielectric parameters, as well as calorimetric measurements. Electrical properties were measured using the four-point method in the frequency range of 4 Hz to 8 MHz and in the temperature range of 30 °C to 50 °C, in 10 °C increments. A total of 1000 measurements were made over the entire frequency range. To perform the measurements, an impedance spectroscopy station was built in the frequency range of 4 Hz to 8 MHz and over a temperature range from room temperature (approximately 30 °C) to 50 °C. The measurements were taken using the 4-point method. This method involves the use of four electrodes: two for injecting alternating current into the sample and two for measuring the voltage between them, which eliminates the influence of electrode contact resistance on the measurement result. The station consists of a Hioki 3536 impedance bridge, directly connected to wires with electrodes embedded in ferrofluid. Nanoparticles and water are measured and placed in an Eppendorf immersed in heated water. The temperature is monitored by an external thermometer. A diagram of the measurement station is shown in Figure 1.

### 2.4. Electron Microscopy Studies and Analysis of the Chemical Composition of the Obtained Material

The morphology and composition of the synthesized Fe_3_O_4_ nanopowder were examined using a scanning electron microscope (SEM, Zeiss EVO MA10, Carl Zeiss Microscopy GmbH, Oberkochen, Germany) equipped with an energy-dispersive X-ray spectroscopy (EDS) system (EDAX Octane Elite, APEX™ EDS, EDAX LLC). For SEM observation, the nanopowder was dispersed in isopropyl alcohol (IPA) and a small droplet of the suspension was deposited onto a silicon wafer substrate. Next, the substrates were heated at a temperature of 50 °C for 1 h to evaporate the alcohol and to obtain a uniform particle distribution. The measurements were performed at an accelerating voltage of 15 kV using a secondary electron detector. EDS analysis was conducted to confirm the elemental composition of the Fe_3_O_4_ phase, with the same accelerating voltage, carefully selected to ensure adequate excitation of the Fe and O peaks. The silicon wafer was used as a neutral substrate to avoid signal overlap. Simultaneously, the beam current was increased significantly up to 0.5 nA to achieve the optimal value of deadtime DT and count number, whereas the working distance was kept at an optimal level for the used EDS detector.

### 2.5. Calorimetric Experiments

The heating ability of the MNPs was quantified by the specific absorption rate (SAR) and by the intrinsic loss power (ILP). The SAR is an extrinsic parameter, and hence it is determined by the physical and magnetic properties of MNPs, which in turn depend on the magnetic field amplitude, *H*_max_, and the frequency, *f*. On the other hand, the ILP is an intrinsic parameter, because removing both extrinsic factors, one arrives at the definition of the intrinsic loss power as ILP = SAR/(fH^2^). Even though both SAR and ILP are widely used as comparative design parameters, they are almost always measured in non-adiabatic systems [24]. In this case, based on the assumption that the temperature of the sample is always homogeneous and that the initial slope of the heating curve does not include any losses (the perfect adiabatic condition is valid), the amount of heat generated in the sample can be quantified in terms of the SAR and calculated as follows:

(1) $$SAR=\frac{P}{m_{MNP}}=c\frac{m_{s}}{m_{MNP}}\frac{\Delta T}{\Delta t}=\frac{c}{\varphi }\frac{\Delta T}{\Delta t}$$

where *c* is the specific heat capacity of the ferrofluid, *m*_s_ is the total sample mass, *m*_MNP_ is the mass of MNPs in the sample, *φ* is the concentration of MNPs in the sample, and ΔT/Δt is the heating time.

The calorimetric experiments were carried out with the use of a magneTherm system (nanoTherics, Staffordshire, UK).

### 2.6. Mössbauer Spectroscopy

The magnetic properties of the magnetite powder sample were investigated using Mössbauer spectroscopy (mElaktronika jądrowa, Kraków, Poland). The measurements were carried out at room temperature (RT), and the spectra were collected with a conventional transmission spectrometer operating in a constant-acceleration mode. The isomer shift values are reported relative to α-Fe at RT to ensure consistency and comparability with standard reference data. A detailed spectral analysis was performed to identify and quantify the different magnetic and structural phases present in the sample. The relative proportions of these phases were obtained from the fitted areas of the corresponding spectral components, under the assumption that all phases exhibit equal recoil-free fractions. This approach provides a reliable estimation of the phase distribution within the magnetite powder.

## 3. Results

### 3.1. Preparation and Analysis of Properties of Fe_3_O_4_ and Fe_3_O_4_-Chitosan Composites

The textural parameters of Fe_3_O_4_, chitosan, and Fe_3_O_4_/chitosan composites were determined using nitrogen adsorption–desorption measurements and the BET model (Table 1). Pure Fe_3_O_4_ exhibited a relatively high specific surface area of 76.3 m^2^/g, with a total pore volume of 0.364 cm^3^/g and an average pore diameter of 19.0 nm, indicating a mesoporous structure. In contrast, both chitosan samples were characterized by very low surface areas (3.7 m^2^/g for chitosan 30–100 cP and 4.5 m^2^/g for chitosan 100–300 cP) and small pore volumes (0.012 and 0.013 cm^3^/g, respectively), which is consistent with the dense and non-porous nature of the polymer. The incorporation of chitosan into Fe_3_O_4_ led to a decrease in surface area compared to pristine Fe_3_O_4_, reaching 72.5 m^2^/g for Fe_3_O_4_/chitosan (30–100 cP) and 68.9 m^2^/g for Fe_3_O_4_/chitosan (100–300 cP). A concomitant reduction in total pore volume and average pore diameter was also observed for both composites. These results suggest that the chitosan layer partially blocks the pores of Fe_3_O_4_ and reduces its accessible surface area. Importantly, no micropore volume was detected in any of the materials, confirming that the porosity is dominated by mesopores. The relatively large average pore diameters (15.8–26.3 nm) further support this conclusion. Overall, the BET analysis demonstrates that the chitosan coating decreases the textural parameters of Fe_3_O_4_ while preserving its mesoporous character.

For Fe_3_O_4_, the ζ potential was positive under acidic conditions and decreased monotonically with increasing pH, crossing zero at the isoelectric point (IEP) and becoming negative in alkaline media. This behavior reflects the protonation–deprotonation equilibrium of the surface Fe–OH groups. The IEP of pristine Fe_3_O_4_ was located at approximately pH 6.5–7.0, in good agreement with the literature values (e.g., pH = 6.6 [25]; pH = 6.7 [26]; pH = 6.81 [27].

**Figure 2:**
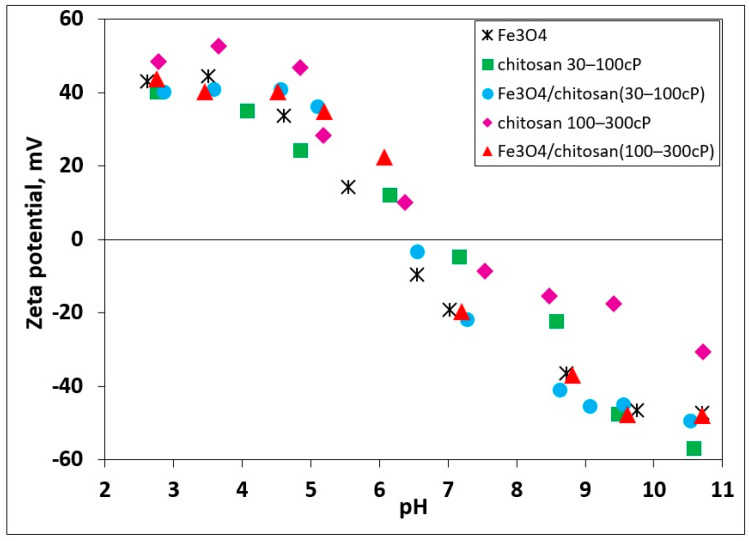
Zeta potential as a function of pH for Fe_3_O_4_, chitosan (30–100 cP and 100–300 cP), and Fe_3_O_4_/chitosan composites in 1 mM NaCl at 25 °C (solid-to-liquid ratio 1:10,000 *w*/*w*).

Both chitosan dispersion series exhibited strongly positive ζ potentials at low pH due to protonated amino groups, followed by a progressive decrease upon basification. Each dispersion displayed an IEP corresponding to amine deprotonation and the reduction of net surface charge. The IEP of chitosan (30–100 cP) was observed at ~pH 7.5, while that of the higher-viscosity chitosan (100–300 cP) was slightly shifted to more alkaline conditions (~pH 8.0). The latter also yielded somewhat higher |ζ| values across the studied pH range, consistent with its higher molecular weight and denser surface coverage (Figure 2).

The Fe_3_O_4_/chitosan composites displayed intermediate behavior between their constituents: ζ remained positive in acidic to near-neutral media due to the chitosan shell, decreased with increasing pH, and crossed zero at composite-specific IEPs. Compared to bare Fe_3_O_4_, the ζ–pH profiles shifted toward more positive values, confirming effective surface coating and charge regulation by chitosan. The IEP of Fe_3_O_4_/chitosan (30–100 cP) was located at ~pH 7.2, whereas Fe_3_O_4_/chitosan (100–300 cP) reached its IEP at ~pH 7.6.

From a colloidal perspective, higher |ζ| values far from the IEP indicate enhanced electrostatic stabilization, while ζ values near zero imply greater aggregation propensity. Among the composites, Fe_3_O_4_/chitosan (100–300 cP) demonstrated superior colloidal stability, as evidenced by its higher positive ζ potentials and more alkaline IEP. This enhanced stability can be attributed to the higher molecular weight of the 100–300 cP chitosan, which provides denser surface coverage and contributes both electrostatic and steric stabilization to the magnetic nanoparticles.

**Figure 3:**
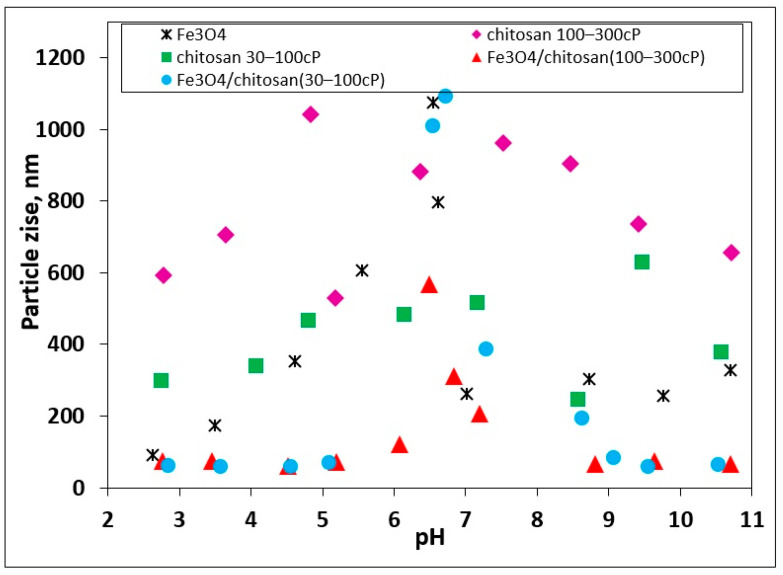
Particle size of Fe_3_O_4_, chitosan (30–100 cP and 100–300 cP), and Fe_3_O_4_/chitosan composites as a function of pH.

The particle size and polydispersity index (PDI) of Fe_3_O_4_, chitosan, and Fe_3_O_4_/chitosan composites were strongly pH-dependent (Figure 3, Table 2). 

For pristine Fe_3_O_4_, particle size was relatively small under acidic conditions (~92 nm at pH 2.6), but increased significantly near the isoelectric point (IEP, pH 6.5–7.0), reaching ~797 nm at pH 6.6, indicative of extensive aggregation. The PDI followed a similar trend, ranging from 0.296 (pH 2.62) to a maximum of 0.586 (pH 6.61), confirming that dispersion uniformity was reduced close to the IEP.

Pure chitosan dispersions showed larger particle sizes across the entire pH range, with strong dependence on molecular weight. For low-viscosity chitosan (30–100 cP), particle sizes varied between ~246 nm (pH 8.6) and ~515 nm (pH 7.2), while the PDI spanned 0.479 (pH 8.58) to 0.719 (pH 9.47), pointing to substantial polydispersity and broad aggregate distributions. In contrast, high-viscosity chitosan (100–300 cP) formed much larger aggregates, exceeding ~960 nm near neutral pH, with PDI values approaching 1.0 (pH 3.65, 4.84, 8.47), indicating highly heterogeneous dispersions with multiple aggregate populations.

The Fe_3_O_4_/chitosan composites demonstrated intermediate behavior between their constituents. For Fe_3_O_4_/chitosan (30–100 cP), particle sizes remained relatively low under acidic conditions (~60–65 nm at pH 2.8–3.6), increased near the IEP to ~385 nm (pH 7.3), and decreased again at alkaline pH. The PDI values ranged from 0.217 (pH 2.85), suggesting highly monodisperse dispersions, to 0.491 (pH 6.73), consistent with aggregation near the IEP. Similarly, Fe_3_O_4_/chitosan (100–300 cP) showed sizes of ~75 nm at acidic pH, swelling to ~567 nm at pH 6.5 before decreasing again under alkaline conditions (~66 nm at pH 10.7). Its PDI values ranged from 0.296 (pH 8.81) to 0.474 (pH 7.20), reflecting moderate polydispersity but overall better dispersion stability compared to pure high-viscosity chitosan.

Taken together, these results confirm that aggregation is most pronounced near the IEPs, where electrostatic repulsion is minimized, while far from the IEP, particle sizes decrease and PDI values improve, indicating enhanced stability. Importantly, the higher-viscosity chitosan (100–300 cP) generally produced larger aggregates and higher PDI values than the 30–100 cP grade, both in pure dispersions and in the composites, which can be attributed to stronger intermolecular interactions, higher molecular weight, and the greater density of functional groups. The particle size measurements were conducted using DLS in an aqueous environment, providing results that are more representative of the conditions relevant to biological systems. The isoelectric point (IEP, ζ = 0) of the examined samples was identified at pH 6.5–7.0. At the IEP, the largest hydrodynamic particle sizes were observed, indicating the formation of aggregates, as the SEM-derived particle sizes were significantly smaller. The aggregation is attributed to particle–particle association, as well as interactions involving chitosan chains and water molecules, including hydrogen bonding among O-H and N-H groups.

### 3.2. X-Ray Diffraction Measurements

**Figure 4:**
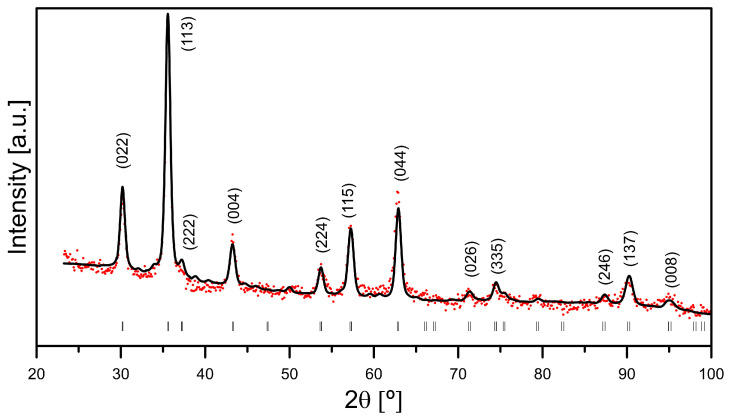
Fitted XRD pattern of magnetite. The red dots are the experimental points, theblack line is the calculated pattern, the vertical lines indicate the angular positions of the peaks characteristic of magnetite.

In order to determine the size of crystallites and crystallographic structure, X-ray diffraction measurements were performed on an Fe_3_O_4_ sample. The diffraction spectrum is shown in Figure 4. The Rietveld method was used to fit the experimental data (red dots) to the theoretical diffractogram profile (black solid line). The markers below denote Miller indices hkl. The peaks in the spectrum correspond to diffraction reflections of magnetite or maghemite (cubic structure with space group *Fd*-*3m*). The obtained spectrum is characterized by significantly broadened lines, which indicates the presence of nanoparticles in the tested sample. The sizes of the nanoparticles determined using the Williamson–Hall formula are 14.3(5) nm. In addition, the nanoparticles are characterized by negligible lattice stresses. The lattice constant determined for Fe_3_O_4_ nanoparticles is 8.361 Å, while the lattice constant of bulk magnetite is 8.397 Å. This seems to be related to the limited size of the nanoparticles, as well as the possibility of partial oxidation of magnetite to maghemite. In this study, we synthesized the materials following the procedure described in [22]. In that publication, the authors measured, calculated, and reported a chitosan-coated thickness of 0.5 nm. We expect that the chitosan coated in our materials is of a similar magnitude.

### 3.3. Measurements of Electrical and Dielectric Properties of Nanoparticles Suspended in Water Using Impedance Spectroscopy

**Figure 5:**
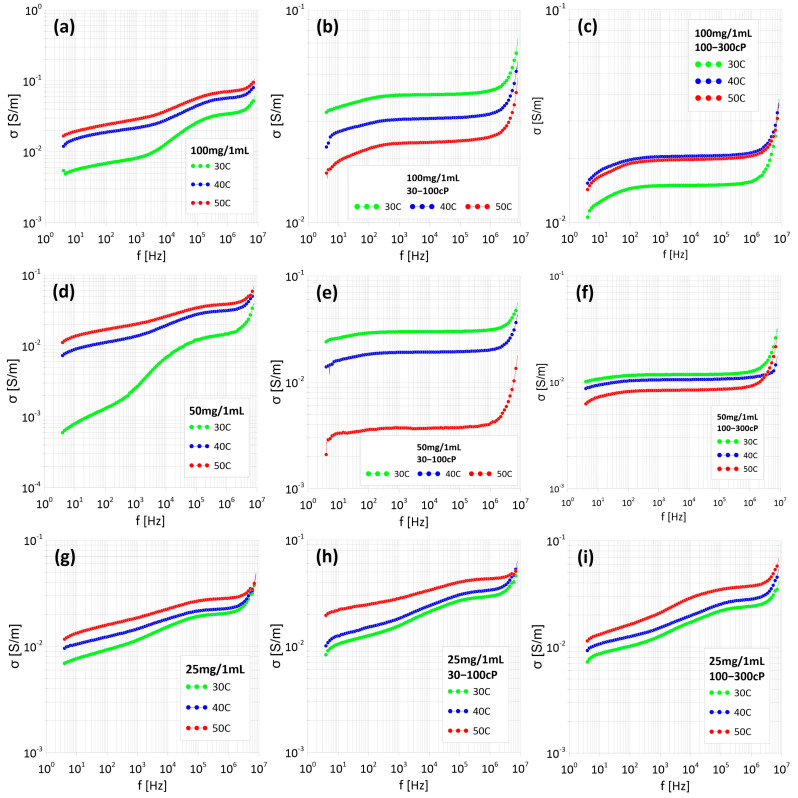
Dependence of conductivity on frequency of samples with different Fe_3_O_4_ nanoparticle content: without chitosan (**a**–**c**), and with chitosan 30-100cP (**d**–**f**) and chitosan 100-300 cP (**g**–**i**).

Measurements of resistance (Rp), capacitance (Cp), phase shift angle (θ), and dielectric loss tangent (tanδ) were carried out as a function of frequency from 4 Hz to 8 MHz and over a temperature range from 30 °C to 50 °C. Figure 5 shows the conductivity measurements of the ferrofluids.

As can be seen in in Figure 5b,e,f, conductivity is practically independent of frequency. Its value decreases with increasing temperature. This type of conductivity curve is characteristic of percolative conduction, with a negative temperature coefficient, and this type of relationship is characteristic of materials with metallic conductivity. In this case, it means that paths have formed between the Fe_3_O_4_ nanoparticles, through which current can freely flow. A different situation can be observed in the other figures, where the conductivity of the material increases with increasing temperature. In this case, we can speak of dielectric conductivity, in which the particles do not interconnect percolation channels and an electron must cross a diffusion barrier to pass from one particle to another. Additionally, it can be seen that the conductivity is significantly lower for samples with nanoparticles encapsulated in the more viscous chitosan.

**Figure 6:**
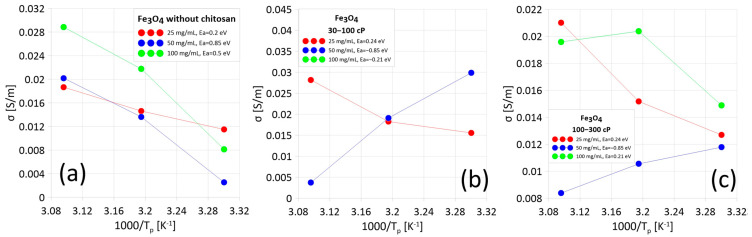
Arrhenius plot with calculated activation energies for suspensions: without chitosan (**a**), with chitosan 30–100 cp (**b**), and with chitosan 100–300 cP (**c**).

Figure 6 presents Arrhenius plots, based on which the activation energy associated with a single electron jump between two potential wells was determined. Analysis of these characteristics revealed distinct differences in the behavior of the studied suspensions. In the case of systems in which electrical conductivity increased with increasing temperature, the obtained activation energy values are positive. These results indicate the dominance of electron tunneling—a process in which electrons overcome a potential barrier, requiring sufficient energy for classical “hopping”. In contrast, in samples for which a decrease in conductivity with increasing temperature was observed, the activation energy turned out to be negative. This type of relationship is typical of conductive materials in which charge movement occurs via classical electron transport. This indicates that continuous percolation paths have formed between the Fe_3_O_4_ nanoparticles, allowing for the free flow of electric current without the need for tunneling mechanisms.

**Figure 7:**
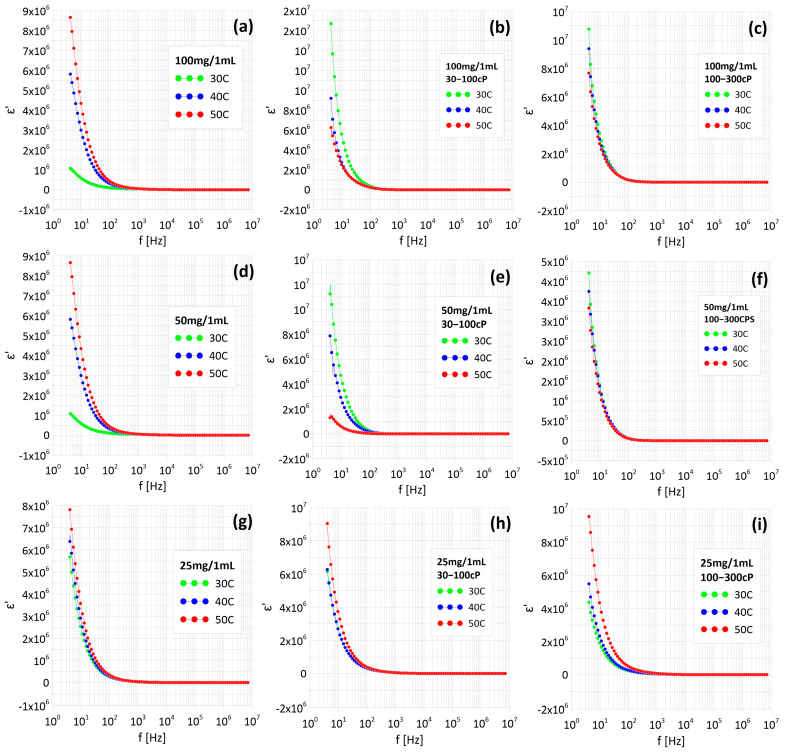
Dependence of real components of the complex dielectric permittivity on frequency of samples with different Fe_3_O_4_ nanoparticle content: without chitosan (**a**–**c**), and with chitosan 30–100 cP (**d**–**f**) and chitosan 100–300 cP (**g**–**i**).

Figure 7 shows the dependence of the true component of dielectric permittivity on frequency. As can be seen, the value of ε’ decreases with temperature and frequency up to 100 Hz in each case. The dominant mechanism responsible for the decreasing true component of dielectric permittivity in Fe_3_O_4_/water ferrofluids is Maxwell–Wagner–Sillars interfacial polarization. This mechanism occurs at the interface between conductive Fe_3_O_4_ nanoparticles and the insulating medium (water), where charges accumulate at the interfaces. Charge transfer and accumulation at the interfaces can result from charge injection from external electrodes, leading to the formation of depleted regions in the insulating medium due to Coulomb blocking. This state persists until the accumulated charges are released—which can occur through tunneling or ohmic conduction if the particles are close together. This phenomenon is associated with the potential formation of nanocapacitors. These structures are formed by adjacent conductive Fe_3_O_4_ nanoparticles separated by an extremely thin layer of oxide or chitosan [28].

**Figure 8:**
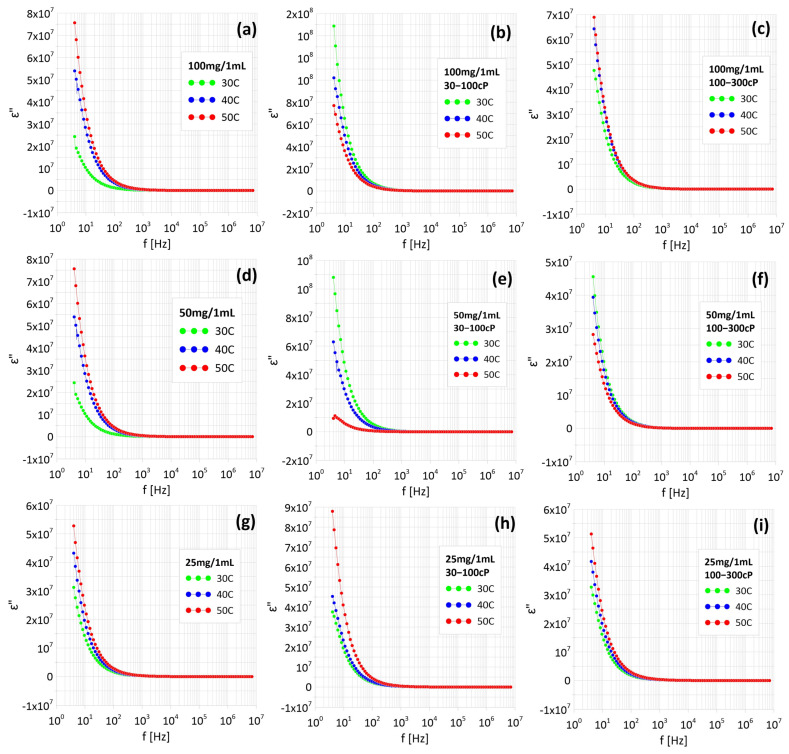
Dependence of imaginary relaxation components of the complex dielectric permittivity on frequency of samples with different Fe_3_O_4_ nanoparticle content: without chitosan (**a**–**c**), and with chitosan 30–100 cP (**d**–**f**) and chitosan 100–300 cP (**g**–**i**).

Figure 8 shows the dependence of the imaginary component of dielectric permittivity as a function of frequency. In all cases, it decreases with increasing frequency. In the samples in which constant current conduction was observed (Figure 5b,e,f), the value of ε” decreases with the measurement temperature. In the sample in which the tunnel conduction mechanism was observed, the value of ε” increases with the temperature. In the rest of the samples, the imaginary part of the permittivity (ε”) remains very high—on the order of hundreds—throughout the entire temperature range studied, up to a frequency of 100 Hz. A similar phenomenon was observed in the samples, whereby the elevated ε” values persisted up to a higher frequency limit of approximately 1 kHz. Such high ε” values in the low frequency range clearly indicate the dominant contribution of conduction losses in the ferrofluid, which are associated with the movement of electric charges within the matrix of the colloidal solution containing nanoparticles. With increasing frequency above the above-mentioned thresholds, a sharp decrease in the ε” value is observed, indicating a significant reduction in conduction losses. This indicates that at higher frequencies, ionic conduction processes and the associated energy loss mechanisms become increasingly less significant, and the dielectric response of the ferrofluid is determined to a greater extent by the structural and orientational polarization of the nanoparticles and the layers of surrounding ligands. As a result, in the higher frequency ranges, the ferrofluid behaves in a manner more typical of dielectric systems, with a limited contribution of conduction to the total losses [19].

### 3.4. Morphology and Composition Analysis

**Figure 9:**
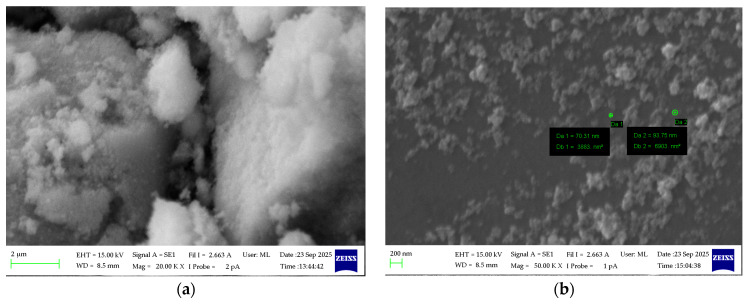
SEM micrographs of the agglomerated Fe_3_O_4_ nanoparticles (**a**) and dispersed Fe_3_O_4_ nanopowder on Si wafer surface (**b**).

The morphology of the synthesized Fe_3_O_4_ nanopowder was examined using SEM at an accelerating voltage of 15 kV, keeping a low beam current. The obtained micrographs, shown in Figure 9a, reveal that the material exhibits a strongly agglomerated structure composed of irregular clusters formed by smaller primary nanoparticles. Such agglomeration is typical for magnetic iron oxide powders and results from interparticle magnetic and surface forces. To evaluate the size of the individual grains, the nanopowder was dispersed on a silicon wafer, which allowed clearer observation of isolated particles. Figure 9b shows the measured particles, and the smallest diameters were found to be in the range of 70–90 nm, confirming the nanometric nature of the synthesized Fe_3_O_4_ material.

**Figure 10:**
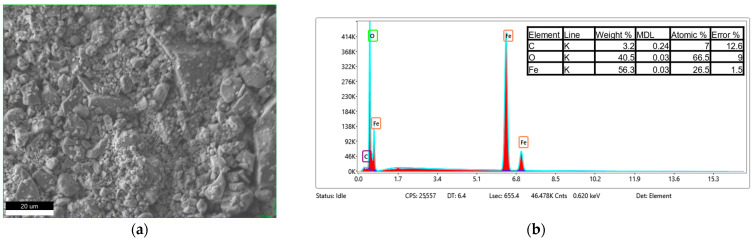
SEM micrograph of the analyzed Fe_3_O_4_ sample (**a**) and corresponding EDS spectrum (**b**).

**Figure 11:**
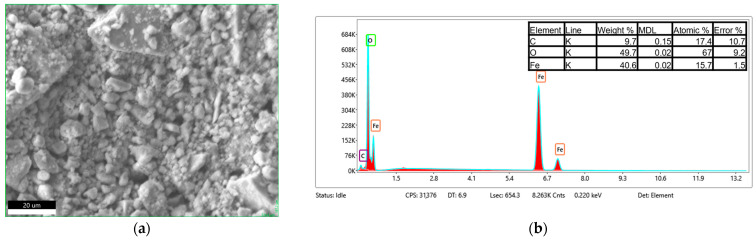
SEM micrograph of the analyzed Fe_3_O_4_/chitosan(30–100 cP) sample (**a**), and corresponding EDS spectrum (**b**).

**Figure 12:**
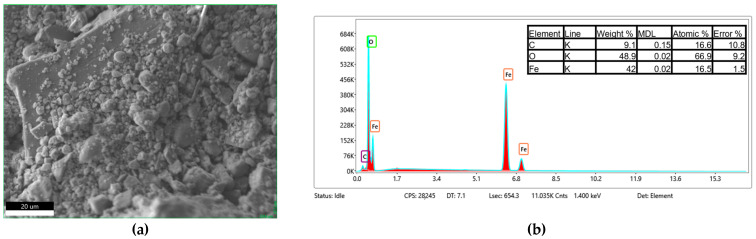
SEM micrograph of the analyzed Fe_3_O_4_/chitosan(100–300 cP) sample (**a**), and corresponding EDS spectrum (**b**).

The EDS analysis, shown in Figure 10, Figure 11 and Figure 12, confirms that the bare Fe_3_O_4_ nanoparticles exhibit a high Fe content with minor surface carbon contamination, while the chitosan-coated samples show a significant increase in carbon content (from ~3 wt.% to ~9 wt.%). Simultaneously, a relative decrease in the detected Fe fraction was observed. This indicates that the chitosan layer effectively covers the nanoparticles and attenuates the Fe signal. Both chitosan types (30–100 cP and 100–300 cP) yield nearly identical EDS compositions, suggesting comparable surface coverage. The O/Fe atomic ratios are higher than the theoretical value, partly due to the organic coating and partly due to systematic EDS limitations for light elements (especially H and N contained in chitosan) and nanopowders.

### 3.5. Magnetic Properties

**Figure 13:**
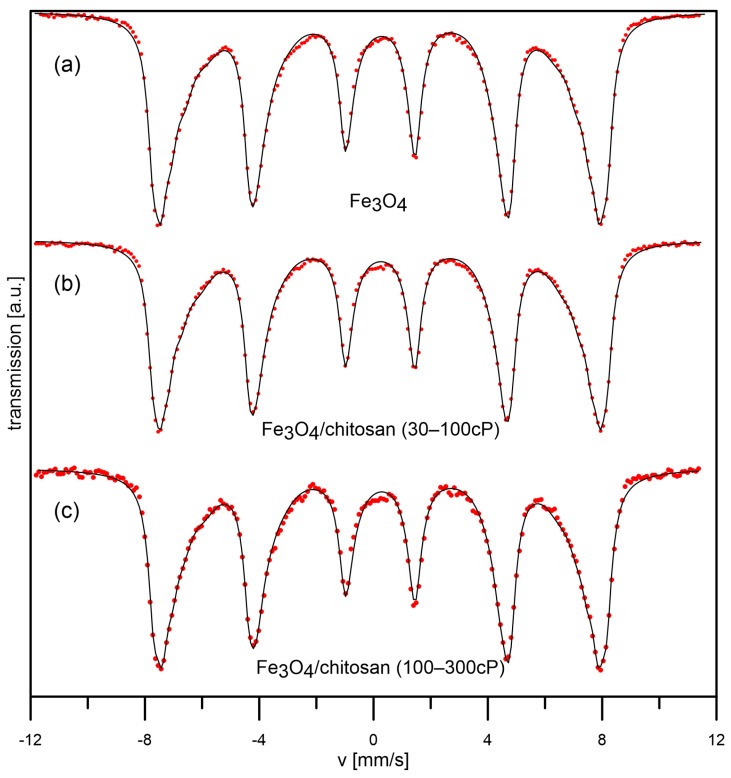
Mössbauer spectra for sample Fe_3_O_4_ (**a**), Fe_3_O_4_/chitosan(30–100cP) (**b**), and Fe_3_O_4_/chitosan(100–300 cP) (**c**) at room temperature.

Mössbauer spectroscopy is an extremely sensitive and convenient method for studying the magnetic properties of various materials. Figure 13 shows the spectra of Fe_3_O_4_ (a), Fe_3_O_4_/chitosan (30–100 cP) (b), and Fe_3_O_4_/chitosan (100–300 cP) samples, taken at room temperature. The Fe_3_O_4_ spectrum consists of a sextet with asymmetric, broadened resonance lines. This spectrum was developed using three ferromagnetic components. The first two sextets are associated with tetrahedral and octahedral iron positions in the magnetite lattice. The third component, with the lowest field value, is characterized by a significant line width. This component may be associated with iron atoms located on the surface of nanoparticles. Atoms on the surface of crystallites do not have a complete environment, which is why magnetic fields and electric field gradients may occur, affecting the line width. On the other hand, as can be observed in the spectrum in question, the resonance lines in the sextet are highly asymmetrical. The outer slopes of the lines are steep, while the lines are broadened on the inside. This may indicate a strong dipole interaction between the nanoparticles. Such an interpretation of Mössbauer spectra of strongly interacting nanoparticles was proposed by Polikarpov and colleagues [29]. Strongly interacting nanoparticles block each other, preventing free oscillations of the resultant magnetization vector of the nanoparticle within a single magnetic domain. This suggests that the nanoparticles are in a state close to the superparamagnetic. The Mössbauer spectra for Fe_3_O_4_/chitosan (30–100 cP) (b) and Fe_3_O_4_/chitosan (100–300 cP) (c) samples differ slightly from the uncoated sample, which may suggest that Fe_3_O_4_ nanoparticle agglomerates were coated.

### 3.6. Measurements Under Non-Adiabatic Conditions

The calorimetric experiments were conducted using 2.0 mL of 100, 50, and 25 mg/mL concentrations of different composites, as indicated in Table 3. The samples were exposed to the same magnetic field, *H*_max_ = 15.9 kA/m with frequency *f* = 532.1 kHz. The experiments were repeated four times for each concentration of the MNPs with fresh samples, while the temperature measurement data were recorded with a resolution of 0.5 s.

**Figure 14:**
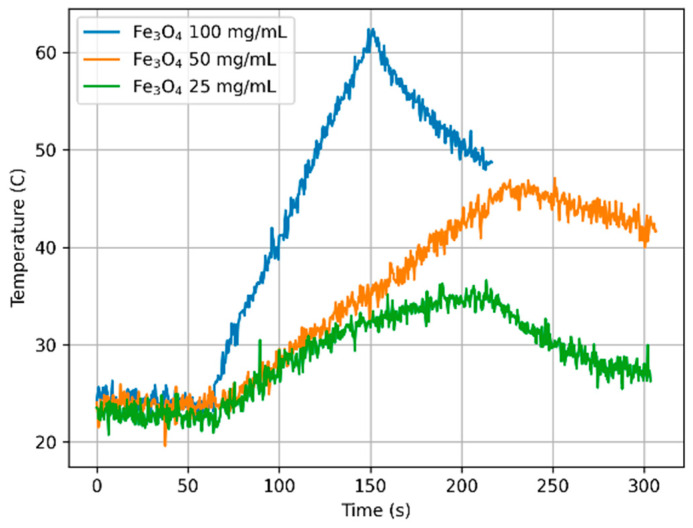
Temperature over time for sample Fe_3_O_4_ with different concentrations (100, 50, and 25 mg/mL), *H*_max_ = 15.9 kA/m, f = 532.1 kHz.

**Figure 15:**
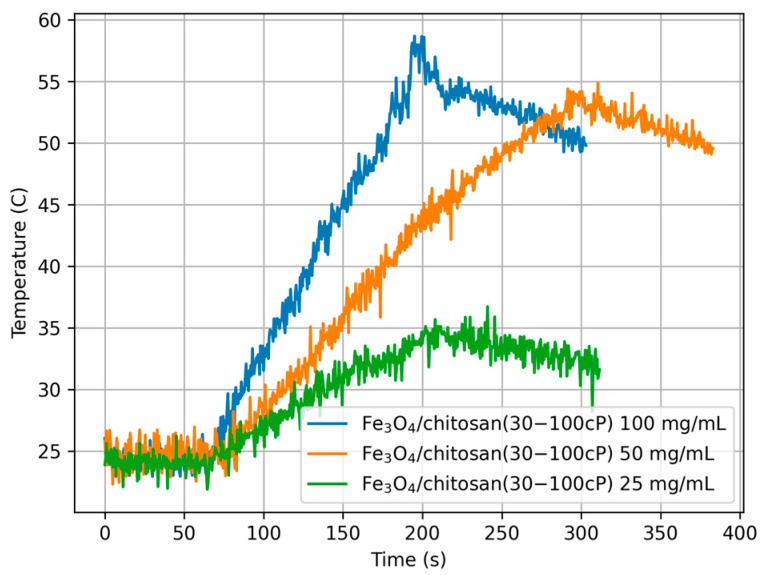
Temperature over time for sample Fe_3_O_4_/chitosan (30–100cP) with different concentrations (100, 50, and 25 mg/mL), *H*_max_ = 15.9 kA/m, f = 532.1 kHz.

**Figure 16:**
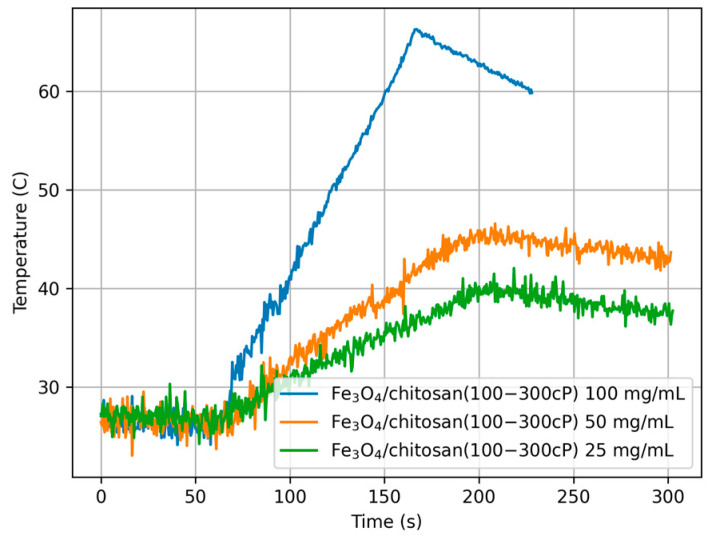
Temperature over time for sample Fe_3_O_4_/chitosan (100–300 cP) with different concentrations (100, 50, and 25 mg/mL), *H*_max_ = 15.9 kA/m, f = 532.1 kHz.

The results of calorimetric temperature measurements as a function of time for the samples are depicted in Figure 14, Figure 15 and Figure 16.

The results of the calculations performed with the use of the aforementioned SLP and ILP estimation methods for the samples are shown in Table 3.

Considering all estimated SAR values, it can be noted that they depend on both the concentration of nanoparticles in the sample and the type of chitosan. The highest SAR values were obtained for the lowest nanoparticle concentration (sample numbers 3, 6, 9, concentration 25 mg/mL), while the lowest values were obtained for sample numbers 1, 4, 7, i.e., at a concentration of 100 mg/mL. It can also be noted that the presence and type of chitosan caused a decrease in SAR values compared to the Fe_3_O_4_ sample, reducing it by 18%, 30%, and 40% for chitosan (30–100 cP) and 12%, 36%, and 17% for chitosan (100–300 cP), respectively.

The above effect, i.e., the influence of concentration and chitosan type on the heating properties of magnetic nanoparticles when the magnetic field and its frequency are constant, can be explained on the basis of linear response theory (LRT) [30]. In calorimetric experiments, the heat dissipation depends on the magnetic field amplitude and its frequency and the physical properties of a sample like the magnetic and hydrodynamic radius of MNPs, solvent viscosity, and saturation magnetization. Besides, particle volume fraction or MNP concentration plays an important role when SAR is estimated.

**Figure 17:**
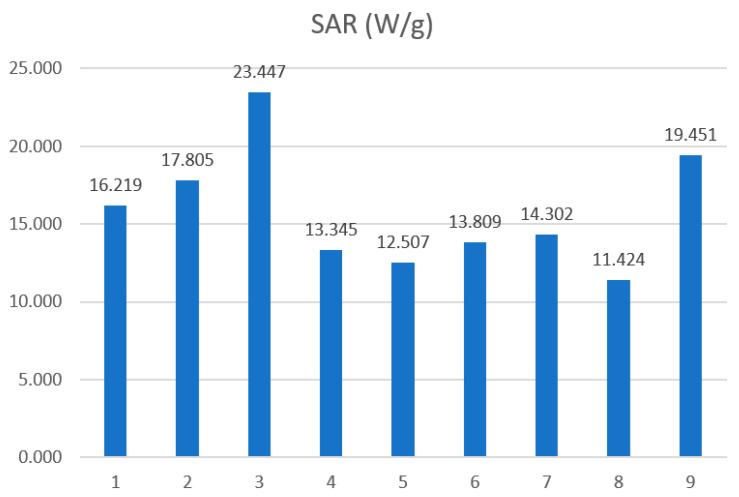
Samples 1, 2, 3 Fe_3_O_4_; 3, 4, 5 Fe_3_O_4_/chitosan (30–100 cP); and 7, 8, 9 Fe_3_O_4_/chitosan (100–300 cP).

Taking into account the results of SAR estimation (see Table 3 and Figure 17), one can conclude that the different fractions of MNPs were rotating in the presence of AMF. It should be mentioned that rotation involves both Neel and Brownian relaxation times $\tau_{N}$ and $\tau_{B}$, respectively. The SAR decreases due to the chitosan coating can be considered taking into account the following formulas:

(2) $$SAR\propto \omega H_{max}^{2}\chi^{\prime \prime }$$

(3) $$SAR\propto \omega H_{max}^{2}\chi_{0}\frac{\omega \tau }{1+{\left(\omega \tau \right)}^{2}}$$

where *H*_max_ is the magnetic field amplitude, ω=2πf is the angular frequency, and $\chi^{\prime \prime }$ is the imaginary part of susceptibility. To include MNP concentration φ, which may increase interparticle interactions as well as clustering, in the above formula, the mean-field approach can be used as follows:

(4) $$\chi_{0,eff}=\frac{\chi_{0}}{1+\alpha \left(\varphi \right)\chi_{0}}$$

where α(φ) indicates the interaction parameter, which increases with particle volume fraction φ. At low φ, it can be assumed that α(φ)∝φ. So, as φ increases, the effective susceptibility $\chi_{0,eff}$ decreases and lowers the SAR. Finally, assuming that τ is constant and that concentration reduces susceptibility by the factor 1/(1+cφ), the SAR can be expressed as follows:

(5) $$SAR\left(\varphi \right)∼\frac{1}{1+c\varphi }$$

So increasing φ reduces the SAR, as indicated in Figure 16 for samples 1, 2, and 3 without chitosan. The same methodology can be applied to chitosan samples, but in this case, the lower SAR can be explained by the different viscosity ranges of the samples compared to water. On the other hand, the heating rate depends on the relaxation time τ and temperature *T.* For Neel relaxation time $\tau_{N},$ a small change in temperature *T* or in $KV_{M}$ produces a huge shift in $\tau_{N}$, according to the following formula:

(6) $$\tau_{N}\left(T\right)=\tau_{0}\exp\left(\frac{KV_{M}}{k_{B}T}\right)$$

where $\tau_{0}$ is the initial time (≈10^−9^), K is the anisotropy constant, $V_{M}$ is the magnetic volume of MNP, and k_B_ is the Boltzmann constant. As for the Brownian relaxation time $\tau_{B}$, it depends on viscosity η(T), temperature, and hydrodynamic radius and can be expressed as

(7) $$\tau_{B}\left(T\right)=\frac{3\eta \left(T\right)V_{H}}{k_{B}T}$$

where $V_{H}={\left(1+\frac{\delta }{R_{M}}\right)}^{3}V_{M}$ is the hydrodynamic volume for a particle of radius $R_{M}$, $V_{M}$ is the magnetic volume, and δ is the thickness of the sorbed surfactant layer. For example, because $V_{H}\sim R_{H}^{3},$ for a larger hydrodynamics radius ($R_{H}$) the Brownian relaxation shifts to lower frequencies, i.e.,

(8) $$f_{B}=\frac{1}{2\pi \tau_{B}}$$

and typically $\tau_{B}$ decreases with increasing temperature *T*, i.e., $k_{B}T$ increases and viscosity decreases. The effects of the coating on Brownian relaxation can be summarized as follows: the thicker the coating, the more the susceptibility $\chi^{\prime \prime }$ peak moves to lower frequencies. As for the relaxation time τ, if the interactions increase τ so that ωτ moves from 1 to values much greater, then

(9) $$\frac{\omega \tau }{1+{\left(\omega \tau \right)}^{2}}\approx \frac{1}{\omega \tau }$$

This means that the SAR falls as 1/t. It should also be underlined that a small change in temperature *T* or in KV produces a huge shift in $\tau_{N}$. For example, the heating properties of MNPs at a fixed particle concentration (samples (1, 4, 7) (2, 5, 8), and (3, 6, 9)) depend on the solvent viscosity, which is related to Brownian relaxation. Moreover, chitosan changes the hydrodynamic radius of MNPs, contributing to SAR (samples 4 to 9). On the other hand, if concentration increases, MNPs can form clusters or aggregates and distort the applied magnetic field and hinder the ability of individual magnetic moments to follow the alternating field efficiently (Néel relaxation). In addition, when Néel relaxation dominates, i.e., for a very viscous solvent, the Brownian contribution is blocked, and the SAR decreases.

## 4. Conclusions

The present study demonstrates that coating Fe_3_O_4_ nanoparticles with chitosan significantly affects both their textural and electrokinetic properties. XRD studies confirm that nanoparticles with an Fe_3_O_4_ core and an average size of 14.3(5) nm have been successfully synthesized using the co-precipitation method. Nanoparticles of this size are suitable for research into the hyperthermia of magnetic liquids. Pure Fe_3_O_4_ exhibited a high specific surface area and mesoporous structure, while chitosan coatings reduced the surface area and pore volume, confirming partial pore blockage. The zeta potential measurements indicated that chitosan effectively shifted the IEPs of the composites toward more positive values, enhancing colloidal stability, especially for the higher-viscosity chitosan (100–300 cP). Particle size and PDI analyses revealed that aggregation was most pronounced near the IEPs, while at pH values far from the IEPs, the dispersions were more stable and monodisperse. Notably, Fe_3_O_4_/chitosan (100–300 cP) displayed better dispersion stability and higher ζ values than Fe_3_O_4_/chitosan (30–100 cP), likely due to its higher molecular weight and denser surface coverage. Overall, the results confirm that chitosan coating can be tuned to modulate the surface charge, particle size, and colloidal stability of Fe_3_O_4_ nanoparticles, which is crucial for applications in nanocomposites, catalysis, and electromagnetic materials. The findings provide insight into the design of Fe_3_O_4_-based nanomaterials with controlled aggregation and enhanced functional properties. 

Two different conduction mechanisms were observed in ferrofluids. In some samples, metallic conductivity dominated, indicating the formation of percolation channels between nanoparticles. In the remaining samples, dielectric conductivity was observed (increasing with temperature), where electrons must overcome diffusion barriers. The actual permittivity component (ε’) decreases with increasing temperature at frequencies up to 100 Hz, which results from the Maxwell–Wagner–Sillars interface polarization occurring at the interface between the conductive nanoparticles and water. The imaginary component (ε”) remains high (in the order of hundreds) at low frequencies (up to 100 Hz–1 kHz), indicating a dominant contribution of conductive losses. Chitosan significantly reduces the conductivity of the material due to its increased viscosity, which limits the mobility of nanoparticles and hinders the formation of percolation channels. Higher chitosan concentrations result in a greater reduction in conductivity. At low frequencies, ionic conduction processes and energy losses dominate. Additionally, it can be seen that in the samples in which the three lowest SAR values were observed, metallic conduction occurs, i.e., electrical paths are formed between nanoparticles. The results demonstrate the possibility of precisely controlling the properties of ferrofluids by modifying their composition and structure. The types of conductivity observed in the samples make them potentially useful for applications requiring stable conductivity, such as magnetic hyperthermia.

As far as calorimetric experiments are concerned, it has been demonstrated that the developed magnetic nanoparticles have the potential for use in magnetic hyperthermia. However, the selection of parameters such as nanoparticle concentration or the amplitude and frequency of the external magnetic field requires further research in order to optimize the SAR. In this context, combining the results of dielectric spectroscopy and calorimetric experiments will allow the above-mentioned parameters to be optimized for medical applications, since the difference between whether the relaxing dipoles are magnetic moments or electric dipoles may play a key role in planning magnetic hyperthermia therapy in vivo or in silico. Therefore, anything that changes relaxation time, like viscosity, temperature, anisotropy, interactions, or clustering, directly shifts the loss peak by the inverse of that change.

## Figures and Tables

**Table 1:** Textural parameters of Fe_3_O_4_, chitosan, and Fe_3_O_4_/chitosan composites determined from N_2_ adsorption–desorption isotherms (BET model).

Sample	Specific Surface Area, m^2^/g	Pore Volume, cm^3^/g	Micropore Volume, cm^3^/g	Pore Diameter, nm
Fe_3_O_4_	76.3	0.364	none	19.0
chitosan 30–100 cP	3.7	0.023	none	25.0
chitosan 100–300 cP	4.5	0.030	none	26.3
Fe_3_O_4_/chitosan (30–100 cP)	72.5	0.286	none	15.8
Fe_3_O_4_/chitosan (100–300 cP)	68.9	0.325	none	18.9

none: the instrument software produced a negative value of micropore volume, which does not have a physical sense.

**Table 2:** The highest and lowest polydispersity index values of the measured dispersions, including the pH value.

Dispersion Series	Minimum Value of the PDI	Maximum Value of the PDI
Fe_3_O_4_	0.296 (pH = 2.62)	0.586 (pH = 6.61)
chitosan 30–100 cP	0.479 (pH = 8.58)	0.719 (pH = 9.47)
chitosan 100–300 cP	0.705 (pH = 9.41)	1 (pH = 3.65, 4.84, 8.47)
Fe_3_O_4_/chitosan (30–100 cP)	0.217 (pH = 2.85)	0.491 (pH = 6.73)
Fe_3_O_4_/chitosan (100–300 cP)	0.296 (pH = 8.81)	0.474 (pH = 7.20)

**Table 3:** SAR and ILP values assessed on the basis of the initial slope method for frequency f = 532 kHz and magnetic field *H*_max_ = 15.9 kA/m.

Sample	Concentration φ mg/mL	SAR W/g	ILP nHm^2^kg^−1^
Fe_3_O_4_	100	16.219	0.120
	50	17.805	0.132
	25	23.447	0.174
	100	13.345	0.099
Fe_3_O_4_/chitosan (30–100 cP)	50	12.507	0.093
	25	13.809	0.102
	100	14.302	0.106
Fe_3_O_4_/chitosan (100–300 cP)	50	11.424	0.085
	25	19.451	0.145

## Data Availability

The original contributions presented in this study are included in the article. Further inquiries can be directed to the corresponding author.
